# Micropeptide AF127577.4-ORF hidden in a lncRNA diminishes glioblastoma cell proliferation via the modulation of ERK2/METTL3 interaction

**DOI:** 10.1038/s41598-024-62710-y

**Published:** 2024-05-27

**Authors:** Baoshun Du, Zheying Zhang, Linlin Jia, Huan Zhang, Shuai Zhang, Haijun Wang, Zhenguo Cheng

**Affiliations:** 1 Department of Neurosurgery, Xinxiang Central Hospital, Xinxiang, 453003 Henan People’s Republic of China; 2 Department of Pathology, Xinxiang Medical University, No. 601 Jinsui Avenue, Xinxiang, 453003 Henan People’s Republic of China; 3 Department of Critical Care Medicine, The Second Affiliated Hospital of Henan University of Science and Technology, Luoyang, 450053 Henan People’s Republic of China

**Keywords:** Micropeptide, lncRNA AF127577.4, METTL3, ERK2, Glioblastoma, CNS cancer, Oncogenes, Tumour-suppressor proteins

## Abstract

Micropeptides hidden in long non-coding RNAs (lncRNAs) have been uncovered to program various cell-biological changes associated with malignant transformation-glioblastoma (GBM) cascade. Here, we identified and characterized a novel hidden micropeptide implicated in GBM. We screened potential candidate lncRNAs by establishing a workflow involving ribosome-bound lncRNAs, publicly available MS/MS data, and prognosis-related lncRNAs. Micropeptide expression was detected by western blot (WB), immunofluorescence (IF), and immunohistochemistry (IHC). Cell proliferation rate was assessed by calcein/PI staining and EdU assay. Proteins interacted with the micropeptide were analyzed by proteomics after co-immunoprecipitation (Co-IP). We discovered that lncRNA AF127577.4 indeed encoded an endogenous micropeptide, named AF127577.4-ORF. AF127577.4-ORF was associated with GBM clinical grade. In vitro, AF127577.4-ORF could suppress GBM cell proliferation. Moreover, AF127577.4-ORF reduced m6A methylation level of GBM cells. Mechanistically, AF127577.4-ORF diminished ERK2 interaction with m6A reader methyltransferase like 3 (METTL3) and downregulated phosphorylated ERK (p-ERK) level. The ERK inhibitor reduced p-ERK level and downregulated METTL3 protein expression. AF127577.4-ORF weakened the stability of METTL3 protein by ERK. Also, AF127577.4-ORF suppressed GBM cell proliferation via METTL3. Our study identifies a novel micropeptide AF127577.4-ORF hidden in a lncRNA, with a potent anti-proliferating function in GBM by diminishing METTL3 protein stability by reducing the ERK2/METTL3 interaction. This micropeptide may be beneficial for development of therapeutic strategies against GBM.

## Introduction

As an extremely fatal malignancy, glioblastoma (GBM) has become one of the most challenging cancers due to its highly refractory to therapies (chemoradiotherapy and immunotherapy) and grim prognosis (2-year survival of about 25%)^1,2^. Despite advanced knowledge in molecular etiology and pathology and the clinical trials in multiple experimental drugs, the patients of GBM remain unprofited for recent years^1,3^.

In order to develop innovatively efficient treatments against GBM, numerous studies have focused on investigating epigenetic modulators that participate in GBM malignant process^4,5^. Among these modulators, long non-coding RNAs (lncRNAs), previously annotated as transcripts lacking protein-coding capacity, have been proved to program a variety of cell-biological alterations associated with malignant transformation-GBM cascade^6,7^. Lately, with improved sequencing methodologies, some lncRNAs have been revealed to harbor short open reading frames (ORFs) (usually < 300 nucleotides) that possess the ability to encode hidden micropeptides^8,9^. These hidden micropeptides have modulatory activities that are implicated in a variety of cellular processes^10^. More intriguingly, these hidden micropeptides can serve as new cancer biomarkers, participators, and therapeutic targets^11,12^.

Here, we report that the ORF (location of lncRNA: 1483–1569 bp) of AF127577.4, which was previously baptized as a lncRNA, can generate a 29-aa endogenous micropeptide AF127577.4-ORF. AF127577.4-ORF functions as a suppressor of GBM cell proliferation in vitro by diminishing the stability of m6A writer methyltransferase like 3 (METTL3), a pro-tumorigenic player in GBM by elevating RNA m6A methylation^13,14^, via its modulation of the ERK2/METTL3 interaction. The novel micropeptide hidden in lncRNA AF127577.4 may be beneficial for development of therapeutic strategies in GBM.

## Results

### Prediction of the coding potential of GBM-related lncRNAs

The hidden polypeptides generated by lncRNAs have emerged as critical players in tumor biology^15^. As a result, we here sought to identify potential micropeptides hidden in GBM-associated lncRNAs. To address this, we downloaded Ribo-seq dataset GSE129757 of LN308 GBM cells from GEO database and obtained 3607 ribosome-bound lncRNAs (count > 0) after ensembol search tool for sequence acquirement. We then searched these presumable lncRNAs for hypothetical ORFs using EMBOSS getORF tool, excluded the sequences with less than 15 nucleotides, and ended up with 46,678 hypothetical ORFs and their respective aa sequences, which we called the generated hypothetical lncRNA-encoded micropeptide FASTA database. Meantime, the proteomics analysis raw files (PXD013541) of LN308 GBM cells, which were based on the MS/MS, were obtained from the database PRoteomics IDEntifications. The obtained raw files were subsequently processed by MaxQuant software using the generated hypothetical micropeptide FASTA database. From this, we discovered 84 hypothetical micropeptides (Supplementary Table S1) hidden in 32 lncRNAs (Supplementary Table S2).

**Figure 1:**
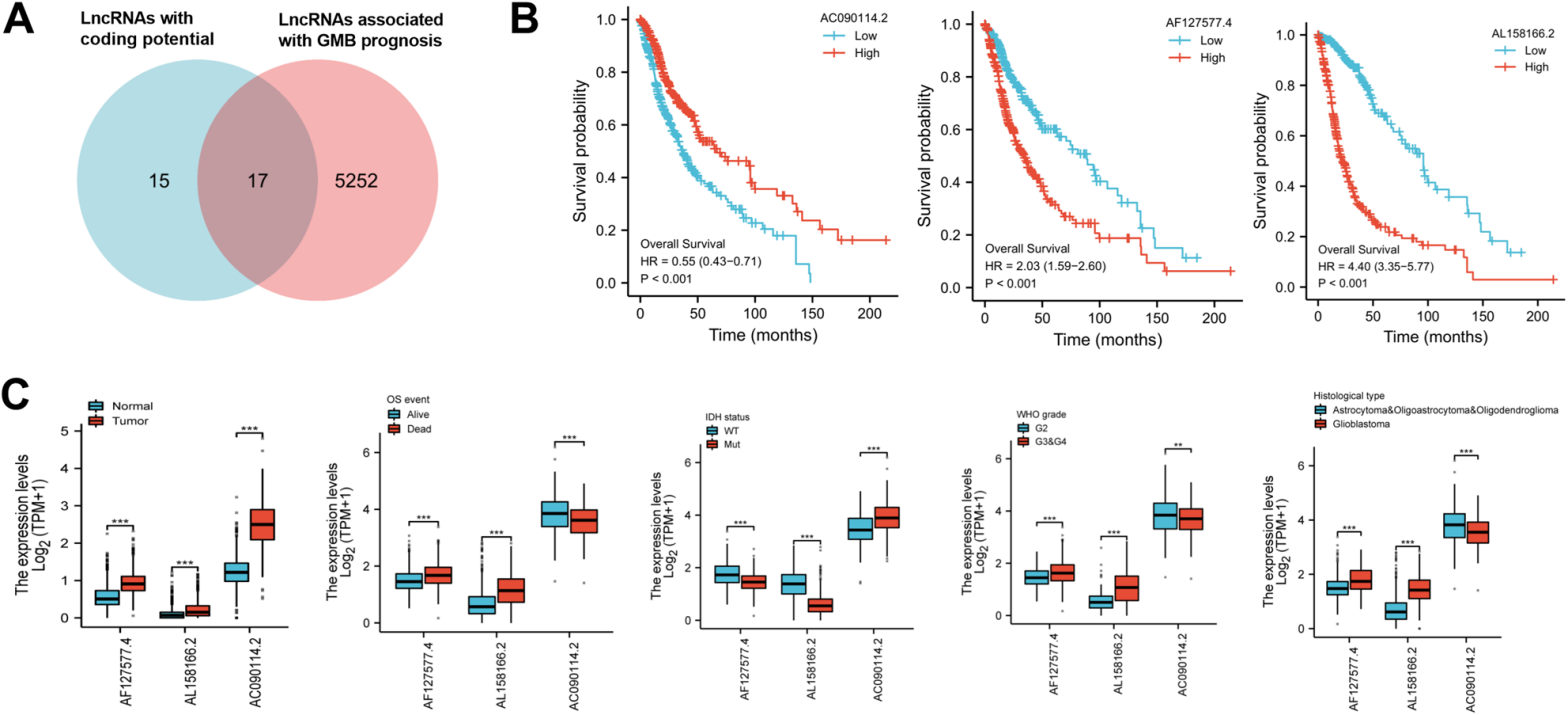
Prediction of the coding potential of GBM prognosis-associated lncRNAs. (**A**) Venn diagram revealed 7 lncRNAs that were correlated with GBM prognosis and were predicted to have the ability to encode protein. (**B**) Association of AC090114.2, AL158166.2 or AF127577.4 expression and GBM poor prognosis. (**C**) Expression of AC090114.2, AL158166.2 and AF127577.4 in GBM and correlation between their expression with overall survival (OS), IDH status, WHO grade and histological type. **P < 0.01, ***P < 0.001.

To further identify GBM prognosis-related micropeptides hidden in lncRNAs, we then retrieved lncRNAs that were related to poor prognosis of GBM patients from GBM RNA-seq data from TCGA database. A total of 5269 lncRNAs was identified by using R survival package (Supplementary Table S3). Next, we combined the obtained 32 lncRNAs with coding potential and 5269 lncRNAs associated with GBM poor prognosis and discovered 17 common lncRNAs (Supplementary Table S4), as illustrated by Venn diagram (Fig. 1A). Based on the inclusion criteria that the optimal length of micropeptide is generally 25–100 aa, a total of 7 lncRNAs were screened (Supplementary Table S5). Among these transcripts, we found that 3 prognosis-related lncRNAs AC090114.2, AL158166.2 and AF127577.4 (Fig. 1B) that were aberrantly expressed in GBM tumors and had close relevance to overall survival (OS), IDH status, WHO grade and histological type (Fig. 1C).

### Identification of AF127577.4-encoded micropeptide AF127577.4-ORF

**Figure 2:**
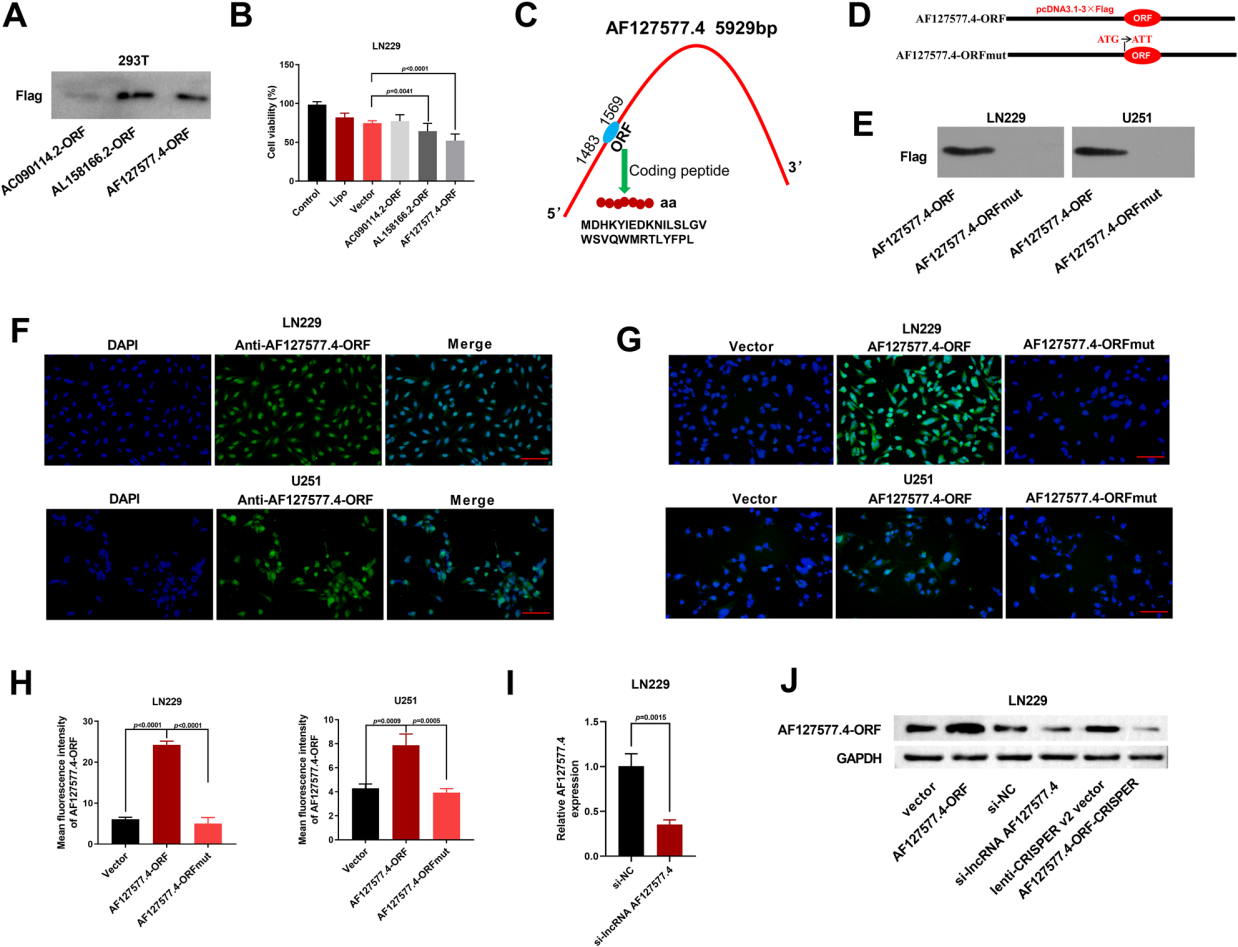
The AF127577.4 ORF encodes a micropeptide AF127577.4-ORF. (**A**) Expression confirmation of AC090114.2-ORF, AL158166.2-ORF and AF127577.4-ORF in 293 T cells by anti-Flag antibody. (**B**) Viability of LN229 cells transfected as indicated was scored by CCK8 method. (**C**) Schematic of the AF127577.4 ORF and its coding peptide sequence. (**D**) Schematic of construction of AF127577.4-ORF and AF127577.4-ORFmut plasmids. (**E**) Detection of the AF127577.4-ORF-Flag fusion peptide in GBM cells after introduction by AF127577.4-ORF or AF127577.4-ORFmut plasmid by IB immunoblotted with anti-Flag antibody. (**F**) Detection of the endogenous AF127577.4-ORF in LN229 and U251 cells by IF microscopy using anti-AF127577.4-ORF antibody. Scale bars: 100 μm. (**G**, **H**) Representative IF images revealing the expression of AF127577.4-ORF in GBM cells transfected as indicated with anti-AF127577.4-ORF antibody. Scale bars: 100 μm. (**I**) RT-qPCR was employed to determine the knockdown efficiency of si-lncRNA AF127577.4 in LN229 cells. (**J**) WB assay was employed to measure the level of AF127577.4-ORF in LN229 cells transfected with vector, AF127577.4-ORF, si-NC, si-lncRNA AF127577.4, lenti-CRISPER v2 vector, and AF127577.4-ORF-CRISPER using its specific antibody.

To confirm if the 3 lncRNAs (AC090114.2, AL158166.2 and AF127577.4) can encode proteins, we created cDNA expression constructs by cloning respective ORF into pcDNA3.1 vector. WB demonstrated the expression of the 3 short ORFs in 293 T cells (Fig. 2A). Given their expression in 293 T cells, we examined the influence of the 3 micropeptides in GBM cell viability. Notably, viability of LN229 GBM cells was clearly impeded by micropeptides AL158166.2-ORF and AF127577.4-ORF (Fig. 2B). AF127577.4-ORF was selected for detailed explanation in this work due to its more significant repression in LN229 cell viability (Fig. 2B). The AF127577.4 transcript with 5929 nucleotides, which was initially annotated as a lncRNA, was found to harbor a ORF with 87 codons (location of lncRNA: 1483–1569 bp, coding a 29-aa micropeptide) (Fig. 2C) that was confirmed to encode a micropeptide (termed AF127577.4-ORF) (Fig. 2A). The AF127577.4 ORF begins in the ATG start codon, which was further validated for its bioactivity by generating the mutation (ATG to ATT) (Fig. 2D). By WB analysis with antibody to Flag tag, AF127577.4-ORF-Flag fusion micropeptide was detected in the AF127577.4-ORF-transfected LN229 and U251 GBM cells, whereas no proteins were detected in the AF127577.4-ORFmut cells (Fig. 2E).

To further demonstrate the endogenous expression of AF127577.4-ORF in GBM cells, we generated a specific antibody to this micropeptide. IF microscopy verified AF127577.4-ORF’s endogenous existence in LN229 and U251 GBM cells (Fig. 2F). Furthermore, in contrast to vector control cells, the cells after introduction by AF127577.4-ORF plasmid showed high expression of this micropeptide (Fig. 2G,H). However, the AF127577.4-ORFmut cells and the control cells were of comparable level of AF127577.4-ORF (Fig. 2G,H). High knockdown efficiency of si-lncRNA AF127577.4 was verified by RT-qPCR (Fig. 2I). We measured the level of AF127577.4-ORF in LN229 cells transfected with vector, AF127577.4-ORF, si-NC, si-lncRNA AF127577.4, lenti-CRISPER v2 vector, and AF127577.4-ORF-CRISPER using its specific antibody by western blot assay. The data showed that the protein level of AF127577.4-ORF was markedly up-regulated upon the transfection of AF127577.4-ORF plasmid, while its level was prominently reduced upon the transfection of si-lncRNA AF127577.4 and AF127577.4-ORF-CRISPER (Fig. 2J), suggesting that AF127577.4-ORF level was regulated by lncRNA AF127577.4. These data together demonstrate the coding ability of AF127577.4 ORF.

### Correlation of AF127577.4-ORF with GBM clinical grade

**Figure 3:**
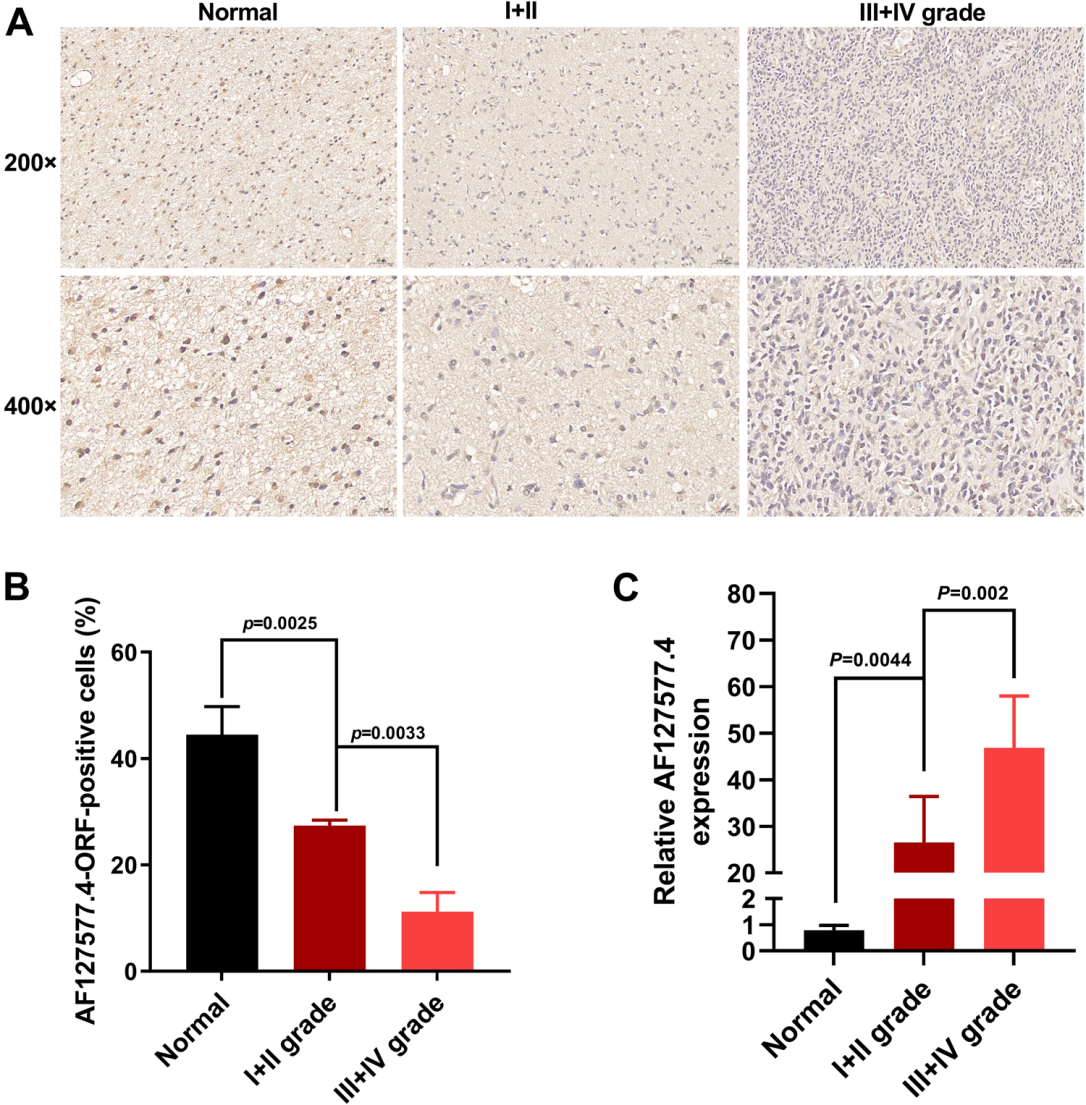
Association between AF127577.4-ORF and GBM clinical grade. (**A**, **B**) IHC showing the expression of AF127577.4-ORF in 3 normal glial tissues, 7 clinical pathological I + II grade tumors, and 10 cases III + IV grade tumors. (**C**) The level of lncRNA AF127577.4 was determined in 3 normal glial tissues, 7 clinical pathological I + II grade tumors, and 10 cases III + IV grade tumors by RT-qPCR.

To examine AF127577.4-ORF’s correlation with clinical pathologic grade, we quantified its levels in normal glial tissues and GBM tumor samples (I + II grade tumors and III + IV grade tumors) by IHC using anti-AF127577.4-ORF rabbit polycolonal antibody. Tumor specimens exhibited fewer cells stained with AF127577.4-ORF than normal controls (Fig. 3A,B). Notably, the positive cells in tumors of III + IV grade were reduced relative to I + II grade tumors (Fig. 3A,B). These results suggest the negative association of AF127577.4-ORF with pathologic grade in GBM. In addition, lncRNA AF127577.4 level exhibited an opposite tendency to micropeptide AF127577.4-ORF (Fig. 3C).

### AF127577.4-ORF suppresses proliferation of GBM cells

**Figure 4:**
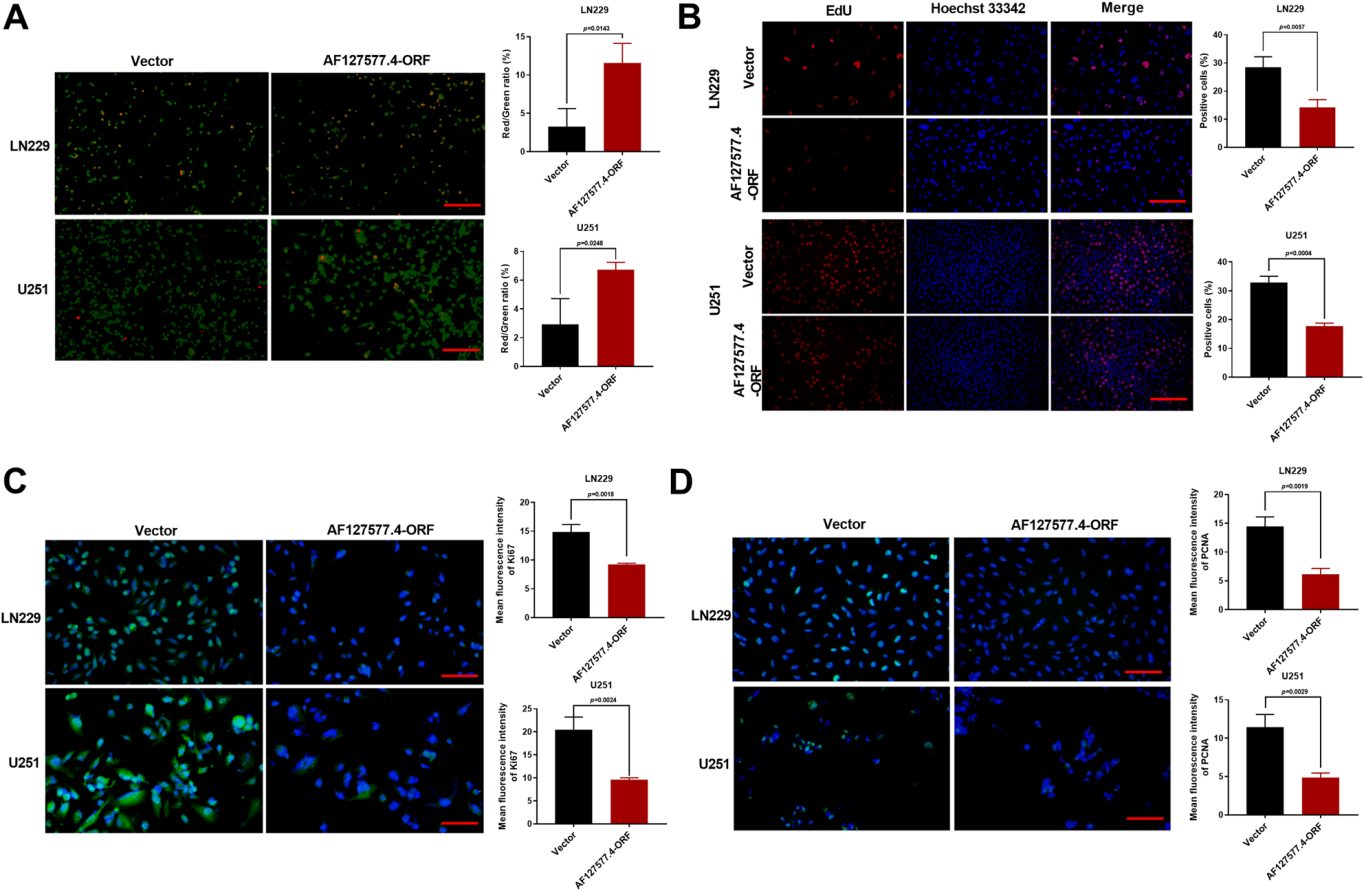
Inhibitory effect of AF127577.4-ORF on cell growth. (**A**) Calcein/PI staining of AF127577.4-ORF- or vector-transfected cells showing cell survival rate. Scale bars: 200 μm. (**B**) EdU analysis of GBM cells transfected as indicated revealing cell proliferation. Scale bars: 100 μm. (**C**, **D**) IF microscopy of AF127577.4-ORF- or vector-transfected GBM cells showing the reduction of Ki67 and PCNA following AF127577.4-ORF increase. Scale bars: 100 μm.

Given the AF127577.4-ORF association with GBM pathological grade, we then asked if the micropeptide could affect GBM cell growth. For these experiments, we elevated the expression of AF127577.4-ORF by the expression construct in LN229 and U251 GBM cells. AF127577.4-ORF increase dramatically induced cell death as measured by the ratio of PI (red)/Calcein (green)-stained cells (Fig. 4A). EdU analysis presented the reduction of the EdU-positive cells after AF127577.4-ORF increase (Fig. 4B), which suggested that AF127577.4-ORF could diminished GBM cell proliferation. We also examined the expression change of Ki67 and PCNA, two proliferating markers, in AF127577.4-ORF-transfected GBM cells. Via IF microscopy, the protein levels of Ki67 and PCNA were strongly downregulated by AF127577.4-ORF (Fig. 4C,D).

### Identification of downstream molecular process of AF127577.4-ORF

**Figure 5:**
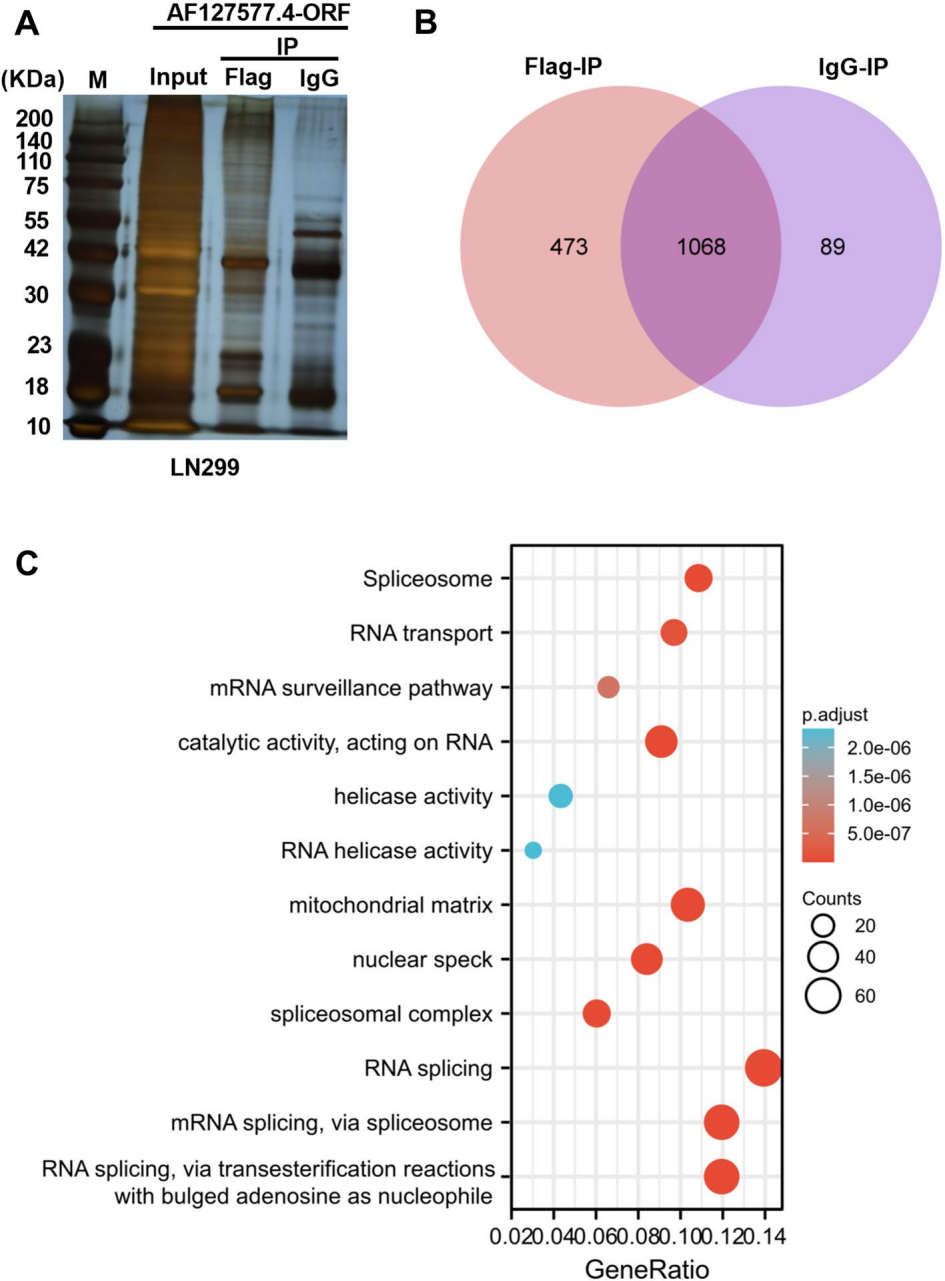
Interacted proteins of AF127577.4-ORF in LN229 cells. (**A**) Silver staining technique after SDS-PAGE of total extractions (Input), proteins pulled down by anti-Flag tag antibody or isotype anti-IgG control. (**B**) Venn diagram displaying the number of the proteins pulled down by anti-Flag tag antibody or isotype anti-IgG control after HPLC/MS. (**C**) The bubble plot revealing the closely associated biological processes and significantly enriched KEGG pathways of the unique proteins pulled down by micropeptide AF127577.4-ORF.

We next investigated molecular determinants by which AF127577.4-ORF participates in GBM process. To address this, we elevated AF127577.4-ORF expression in LN229 GBM cells via the Flag fusion expression construct and carried out Co-IP experiment by using rabbit polyclonal antibody against Flag tag. Silver staining technique after SDS-PAGE revealed a specific band in Flag-containing immunoprecipitates versus isotype anti-IgG controls (Fig. 5A). The subsequent technique of HPLC/MS showed 473 unique proteins coprecipitated by AF127577.4-ORF fused with Flag tag after the exclusion of miscellaneous protein (Fig. 5B and Supplementary Table S6). We then analyzed the potential function of the 473 proteins and their enrichment signaling pathways. Via the enrichment analysis of GO and KEGG pathways, we discovered that these proteins had a tight relationship with spliceosome, RNA transport, RNA splicing, and RNA helicase activity (Fig. 5C). In addition, String tool (https://cn.string-db.org/) showed that these proteins could form a complex regulatory network (Supplementary Fig. S1).

### AF127577.4-ORF diminishes METTL3 stability via the repression of ERK2/METTL3 interaction

**Figure 6:**
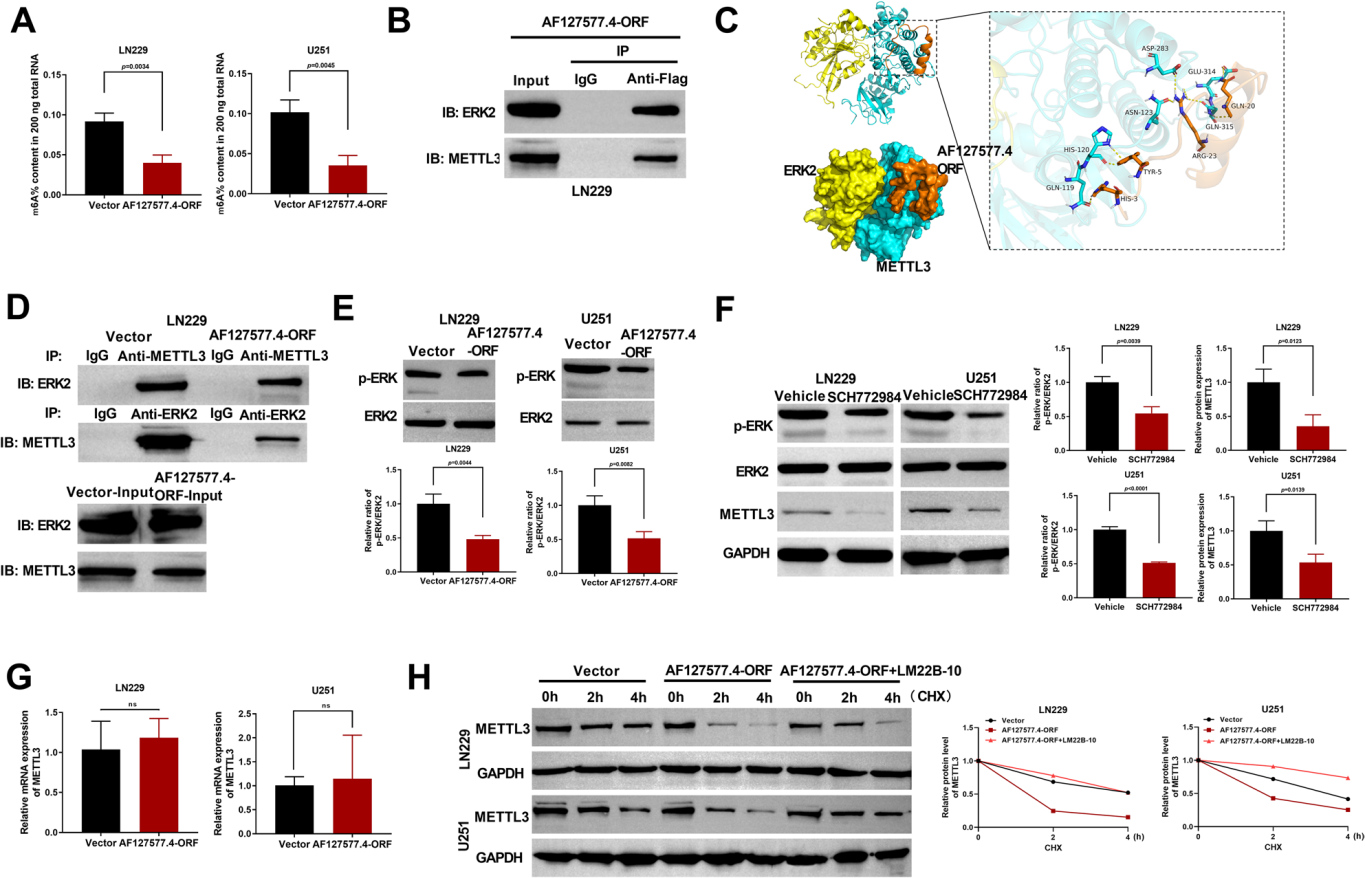
AF127577.4-ORF weakens METTL3 stability via reducing ERK2/METTL3 interaction. (**A**) The mRNA m6A methylation level of LN229 and U251 GBM cells after introduction by AF127577.4-ORF plasmid or vector control. (**B**) IB of Flag-containing immunoprecipitates and IgG controls after Co-IP assays and total cellular lysates (Input) showing the enrichment levels of ERK2 and METTL3. (**C**) Schematic of the predicted AF127577.4-ORF/ERK2/METTL3 complex analyzed using the protein–protein docking poses. (**D**) Co-IP experiments were performed in AF127577.4-ORF plasmid- or control vector-transfected LN229 cells using antibodies against METTL3 or ERK2. Then, the enrichment level of ERK2 or METTL3 was gauged by IB analysis. (**E**) Detection of p-ERK and ERK2 levels in GBM cells transfected as indicated by IB analysis. (**F**) GBM cells were treated with or without SCH772984 and followed by detection of p-ERK, ERK2, and METTL3 levels by IB. (**G**) Real-time qPCR of GBM cells transfected as indicated detected METTL3 mRNA level. H, LN229 and U251 GBM cells transfected as indicated were pre-treated with or without LM22B-10 and then incubated with cycloheximide (CHX) for 0, 2 and 4 h, and followed by detection of METTL3 protein level. ns: no significant.

Among the 406 proteins interacted with AF127577.4-ORF, we found multiple m6A methylation-related proteins. We therefore examined if the micropeptide could affect m6A RNA methylation level of GBM cells. Indeed, we observed a reduction in m6A methylation level in LN229 and U251 cells when introduced with AF127577.4-ORF expression plasmid (Fig. 6A).

In GBM, m6A writer METTL3 has been reported as a pro-tumorigenic player by upregulating target oncogenes via elevating their m6A RNA methylation^13,14^. Moreover, ERK (also called MAPK1) can enhance stability and expression level of METTL3 and thus induces m6A methylation^16^. Intriguingly, ERK2 and METTL3 were found to interact with AF127577.4-ORF in LN229 GBM cells by HPLC/MS proteomic analysis after Co-IP (Supplementary Table S6). WB analysis after Co-IP experiments also confirmed the micropeptide’s interaction with ERK2 and METTL3 (Fig. 6B). We then used ZDOCK 3.0.2 software to predict the protein–protein docking poses and found a complex among AF127577.4-ORF, ERK2 and METTL3, of which AF127577.4-ORF directly bound with METTL3 and METTL3 directly interacted with ERK2 (Fig. 6C). HIS-3, TYR-5, and GLN-20 on AF127577.4-ORF formed hydrogen bonds with GLN-119, HIS-120, and GLU-315/-314 on METTL3, respectively (Fig. 6C). ARG-23 on AF127577.4-ORF formed hydrogen bonds with ASN-123, ASP-283 and GLN-315 on METTL3 (Fig. 6C). These formed hydrogen bonds suggested that the combination of the complex was strong.

In AF127577.4-ORF-upregulating LN229 cells, ERK2 interaction with METTL3 was dramatically weakened relative to vector controls (Fig. 6D). Moreover, AF127577.4-ORF led to a decrease in phosphorylated ERK (p-ERK) level in GBM cells (Fig. 6E), which suggested the inhibitory function of the micropeptide in the activation of the ERK pathway. Previous work has uncovered the enhancement of ERK in METTL3 protein stability and expression level^16^. To verify this finding, SCH772984, a repressor of the ERK pathway, was used to treat the GBM cells. Treatment of SCH772984 significantly diminished p-ERK level of GBM cells (Fig. 6F). Importantly, SCH772984 strongly downregulated METTL3 protein level (Fig. 6F), but it did not change the expression of METTL3 mRNA (Fig. 6G). To examine whether AF127577.4-ORF could affect METTL3 protein stability via ERK pathway, we adopted the protein stability assay of cycloheximide (CHX), a well-known repressor of protein synthesis, in LN229 and U251 GBM cells. Under CHX treatment, AF127577.4-ORF increase led to a striking reduction in METTL3 protein level, whereas the ERK activator LM22B-10 clearly abrogated the repression by AF127577.4-ORF (Fig. 6H), which indicated that AF127577.4-ORF could impair METTL3 stability via the ERK pathway.

### AF127577.4-ORF suppresses GBM cell proliferation via METTL3

**Figure 7:**
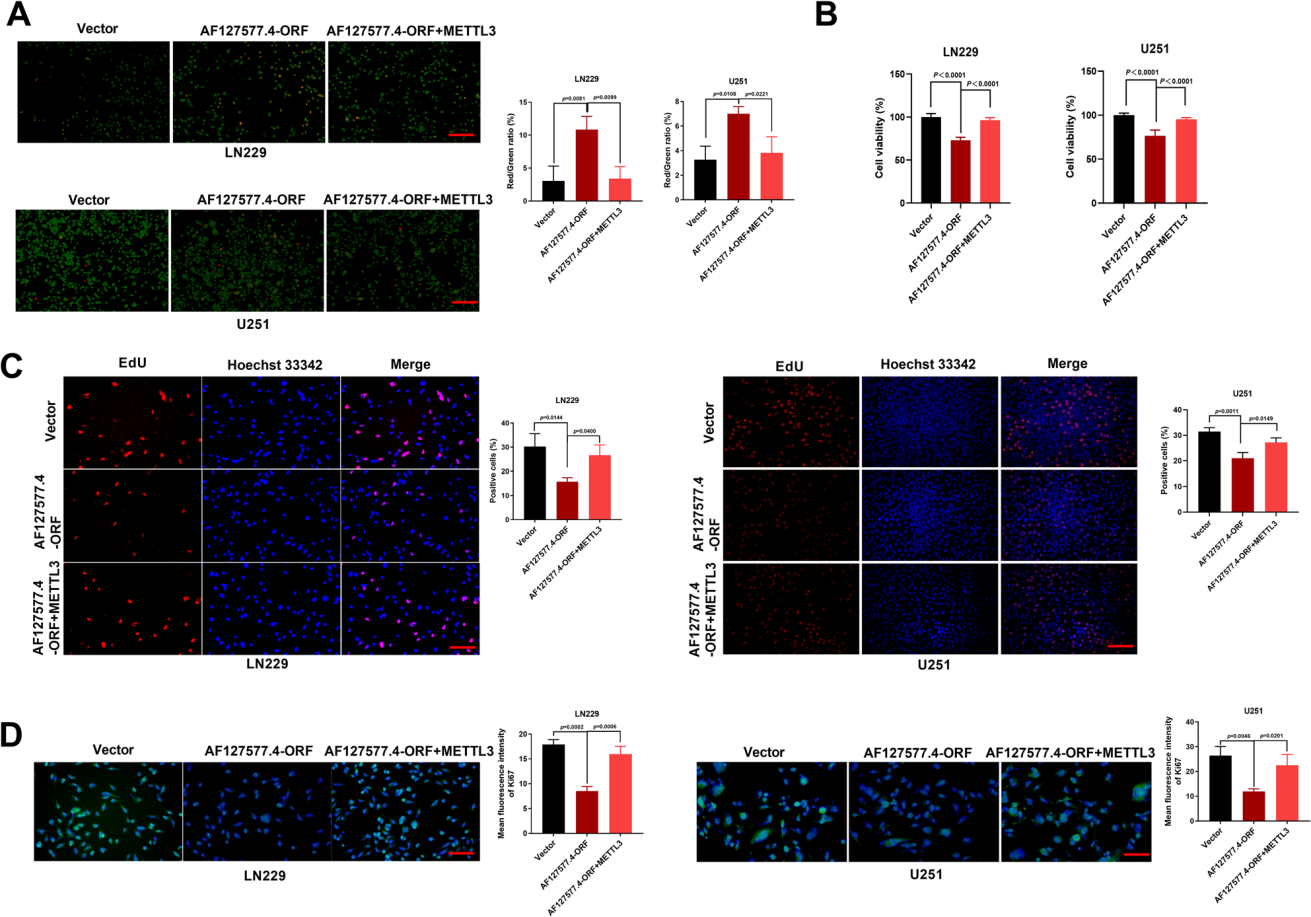
AF127577.4-ORF exerts anti-growth activity in GBM cells via METTL3. (**A**) Calcein/PI staining revealing the death rate (red/green) of LN229 and U251 GBM cells introduced with vector control, AF127577.4-ORF, or AF127577.4-ORF + METTL3 plasmids. Scale bars: 200 μm. (**B**) CCK8 assay was employed to analyze the viability of LN229 and U251 GBM cells introduced with vector control, AF127577.4-ORF, or AF127577.4-ORF + METTL3 plasmids. (**C**) EdU analysis revealing the proliferation rate of GBM cells transfected as indicated. Scale bars: 100 μm. (**D**) IF microscopy showing Ki67 level in transfected GBM cells. Scale bars: 100 μm.

Because METTL3 actively participates in GBM progression^13,14^ and AF127577.4-ORF regulates METTL3, we tested whether METTL3 is responsible for AF127577.4-ORF’s ability to repress cell proliferation. GBM cells were introduced with AF127577.4-ORF plasmid alone or along with METTL3 cDNA construct. Upregulation of METTL3 abrogated AF127577.4-ORF-induced cell death enhancement (Fig. 7A, B) and rescued the micropeptide-imposed proliferation impairment (Fig. 7C). Upregulation of METTL3 also enhanced the expression of Ki67 in AF127577.4-ORF-increased GBM cells (Fig. 7D), which supported the suppression of AF127577.4-ORF in cell growth via METTL3.

## Discussion

By establishing the identification workflow of GBM prognosis-related micropeptides hidden in lncRNAs, we found 3 lncRNAs, which were further confirmed to have the ability to encode a micropeptide. Of them, AF127577.4-ORF was identified as the most significant suppressor of GBM cell viability. Our data also indicated the endogenous expression of AF127577.4-ORF in GBM cells. It is interesting to note that the micropeptide can function as an anti-proliferating protein in GBM cells. The alteration of GBM cell proliferation involves Ki67^17^, which is a well-known marker of proliferation^18^, and PCNA^17^, which is a modulator of DNA synthesis^19^. By determining their expression, we further reinforced AF127577.4-ORF’s anti-proliferative role. Furthermore, IHC analysis of clinical specimens suggested the negative relationship between the micropeptide and GBM clinical grade. It’s worth noting that AF127577.4 lncRNA, which encodes the AF127577.4-ORF, significantly associates with higher GBM grade and reduced patient survival, whilst the AF127577.4-ORF micropeptide encoded by the same lncRNA is negatively correlated to GBM grade and reduces both cell proliferation and ERK activation and also increases cell death. LncRNAs have been reported to exert their functions through serving as RNA molecules or encoded-peptides/proteins, and the roles of lncRNAs and encoded-peptides/proteins might be entirely different. For instance, Ji et al. reported that LINC00665 contributed to breast cancer progression through miR-379-5p/LIN28B axis^20^. Also, Guo et al. reported that micropeptide CIP2A-BP encoded by LINC00665 blocked breast cancer progression^21^. This phenomenon may be related to the independent working mechanisms of lncRNA KDM4A-AS1 and KDM4A-AS1-encoded peptide. In addition, AF127577.4-ORF is a micropeptide encoded by lncRNA AF127577.4, but whether there are other open reading frames of this lncRNA with coding ability remains still unclear. In a recent report, Zhang and colleagues uncovered an 87-aa uncharacterized micropeptide hidden in the circular form of lncRNA LINC-PINT, which hinders GBM cell proliferation by downregulating several oncogenes by its interaction with PAF1c^22^.

m6A methylation, a uniquely crucial way of RNA modification, is capable of influencing the complexity of tumor progression via the modulation of cancer-related biological functions^23^. In light of the m6A research, emerging interests in developing specific anti-tumor therapeutics have focused on inhibitors of m6A writers or erasers^24^. In the further survey of molecular determinants underlying the anti-proliferating role of AF127577.4-ORF using HPLC/MS after Co-IP, we found multiple proteins responsible for m6A methylation. Surprisingly, we uncovered the downregulated effect of AF127577.4-ORF on m6A RNA methylation level of GBM cells. Among these m6A-associated proteins, METTL3 was interesting in our current work due to its role as a m6A writer^25^ and a pro-tumorigenic player in GBM^13,14^. The activation of the ERK pathway has promoting functions in m6A RNA methylation, thereby contributing to GBM tumorigenesis^26^. Furthermore, through physical interaction, ERK stabilizes METTL3 by phosphorylating METTL3 to upregulate and activate METTL3 and thus promotes m6A RNA methylation^16,27^. Through protein–protein interaction (PPI) analysis, we also found the direct relationship of ERK2 and METTL3, both of which were demonstrated to interact with AF127577.4-ORF in GBM cells. Using the analysis of protein–protein docking poses, we predicted a theoretical AF127577.4-ORF/ERK2/METTL3 complex. Our data also pointed out the interference effect of AF127577.4-ORF on ERK2 interaction with METTL3. Activated ERK can directly phosphorylate METTL3 at three residues and enhance METTL3 activity^16,27^. Our findings showed that ERK inactivation can diminish METTL3 protein level. Because AF127577.4-ORF did not affect METTL3 mRNA expression, we first established evidence that AF127577.4-ORF diminishes METTL3 protein stability by suppressing ERK2/METTL3 interaction and inactivating ERK. Additionally, via rescue experiments, we demonstrated the anti-proliferating function of AF127577.4-ORF in GBM cells through METTL3. The regulation of AF127577.4-ORF in METTL3 phosphorylation via ERK will be analyzed in GBM cells in the future.

In GBM, upregulated METTL3 can enhance m6A methylation of ADAR1 mRNA and upregulate its protein level, thereby inducing the occurrence of pro-tumorigenic events^13^. In GBM microvascular endothelial cells, METTL3 promotes CPEB2 mRNA stability through the induction of m6A mRNA methylation and thus regulates the permeability of blood-tumor barrier^28^. The involvement of these target genes of METTL3 in the regulation of the AF127577.4-ORF/ERK2/METTL3 cascade will be deeply explored in the next step. Although we have revealed the association of AF127577.4-ORF level with GBM clinical grade, the precise function of the micropeptide in tumorigenesis and GBM progression remains to be defined using various in vivo experimental models.

Overall, our findings shed light on a novel micropeptide AF127577.4-ORF, which is generated by lncRNA AF127577.4, with a potent anti-proliferating function in GBM by diminishing the protein stability of m6A writer METTL3 by the suppression of the ERK2/METTL3 interaction. The micropeptides hidden in lncRNAs may provide insight for GBM development and could have potential therapeutic values in GBM.

## Methods

### Data retrieving, processing tool, and bioinformatics analysis

The data of ribosome-bound lncRNAs were retrieved from Ribo-seq dataset GSE129757 of LN308 GBM cells from GEO database. We searched their sequences using publicly available ensembol tool (http://asia.ensembl.org/index.html). The hypothetical ORFs of these presumable lncRNAs were predicted by EMBOSS getORF tool (http://emboss.bioinformatics.nl/cgi-bin/emboss/getorf). The mass spectrometry (MS)/MS proteomics analysis raw files (PXD013541) of LN308 GBM cells were downloaded from online database PRoteomics IDEntifications^29^ and processed by MaxQuant software (http://www.maxquant.org). TCGA database containing GBM RNA-seq data was mined to retrieve the lncRNAs related to poor prognosis of GBM patients and the data were processed by R survival package. We assessed the protein–protein interaction using online tool String (https://cn.string-db.org/). Analyses of GO and KEGG pathway enrichment were executed by available database DAVID^30^. We observed the protein–protein docking pose using available tools ZDOCK 3.0.2^31^, Prodigy (https://wenmr.science.uu.nl/prodigy/) for binding energy evaluation, and PyMOL 2.5.4 for visualization.

### Plasmid constructs and siRNAs

To verify their coding abilities, the ORF sequences of AC090114.2, AL158166.2 and AF127577.4 lncRNAs, which were synthesized by Tsingke (Zhengzhou, China), were subcloned in a pcDNA3.1–3 × Flag vector (#BR084, Fenghui Biotechnology, Changsha, China), respectively. A mutant (AF127577.4-ORFmut), in which the AF127577.4 ORF start codon was mutated to ATT, was created by ligating the mutated ORF sequence in the pcDNA3.1-basic vector. SiRNA targeting lncRNA AF127577.4 (si-lncRNA AF127577.4) was synthesized by GenePharma (Shanghai, China). AF127577.4-ORF knockout cell line was generated through CRISPR technology by writegene, and the procedure was as follows: 1. The sgRNA was cloned into the lentiCRISPR-v2 vector. 2. The genome editing efficiency of sgRNA was tested. Human pcDNA3.1-Flag-METTL3 construct (#53,739) was ordered from Miaoling Biology (Wuhan, China).

### Cell culture, treatment, and transfection

LN229 (#STCC11001G) and U251 (#STCC11007P) GBM cell lines were ordered from Servicebio (Wuhan, China). We cultured and maintained them in an atmosphere of 5% CO_2_ at 37℃ in Servicebio-formulated DMEM/High glucose (#G4511) enriched with 10% of FBS (#30,067,334, Thermo Fisher Scientific, New Zealand) and antibiotics (#G4003, Servicebio). To inactivate ERK pathway, 5 μM of its selective inhibitor SCH772984 (#S7101, Selleck, Shanghai, China) was utilized to treat GBM cells for 24 h.

For transfection of GBM cell lines, we applied Lipofectamine 3000 (Invitrogen, Thermo Fisher Scientific) as described elsewhere^32^. In brief, GBM cells were plated at a suitable density to yield approximately 70% confluence at the time of transient transfection (usually at 24 h post-seeding). Lipofectamine reagent and the indicated plasmid(s) were mixed in Opti-MEM media, and followed by addition to each well. Six hours later, fresh regular media were employed to culture cells for 48 h.

### Western blot (WB) for protein analysis

GBM cells after plasmid transfection or SCH772984 treatment were re-suspended in lysis buffer consisted of RIPA buffer (#G2002, Servicebio) and phosphatase/protease inhibitor cocktail (#78,440, Thermo Fisher Scientific) and homogenized by sonication. After denaturation in boiling water for 5 min, SDS-PAGE of protein extracts was executed using 10% or 12% polyacrylamide gels in Tris/Glycine buffer, and followed by transfer onto Immonbilo®-PSQ 0.2 μm PVDF membranes (#ISEQ07850, Millipore, Darmstadt, Germany). For expression detection of lncRNA ORFs, WB was done using the rabbit polyclonal anti-Flag tag antibody (#GB11938-100, Servicebio) at a dilution of 1:1,000. For the detection of ERK2, phosphorylated-EKR (p-ERK), and METTL3, we performed WB analysis with antibodies including anti-ERK2 (rabbit monoclonal, #ab32081, Abcam, Cambridge, UK) at a 1:1,500 dilution, anti-p-ERK (rabbit monoclonal, #ab201015, Abcam) at a 1:1,000 dilution, and anti-METTL3 (rabbit polyclonal, #15,073–1-AP, Proteintech, Wuhan, China) at a 1:1,000 dilution, respectively. The HRP-linked IgG (H + L) (#31,462, Invitrogen) was applied as secondary antibody at a dilution of 1:50,000. Quantification of band intensities were carried out using ImageJ software after visualization through chemiluminescence system (#G2014, Servicebio). Expression of GAPDH (rabbit polyclonal, #10,494–1-AP, Proteintech, 1:10,000), which did not significantly change in this work, was utilized for normalization. The original gel image of the Western blot can be found in the supplementary material raw gel figures document.

### Cell viability assay

LN229 GBM cells grown in a 96-well white TC dish were subjected to transfection of different plasmids. The influence of micropeptides hidden in lncRNAs on LN229 cell viability was evaluated by CCK-8 assay (Cell Counting Kit-8, #96,992, Sigma-Aldrich, Darmstadt, Germany). After incubation by CCK-8 solution for 3 h, formed formazan was quantified by gauging the optical density at 450 nm.

### Preparation of anti-AF127577.4-ORF antibody

The synthesis of micropeptide AF127577.4-ORF and the preparation of anti-AF127577.4-ORF antibody were carried out by Writegene (Zhengzhou, China). In brief, synthesized micropeptide was inoculated into rabbits, and rabbit polyclonal anti-AF127577.4-ORF antibodies were acquired by collecting blood samples and purification by affinity chromatography.

### Immunofluorescence (IF) for AF127577.4-ORF, Ki67, and PCNA

The expression of AF127577.4-ORF, Ki67, and PCNA in un-transfected or transfected GBM cells was evaluated by IF staining with anti-AF127577.4-ORF (1:300 or 1:600), anti-Ki67 (rabbit monoclonal, #MA5-14,520, Invitrogen, 1:250), and anti-PCNA (rabbit polyclonal, #10,205–2-AP, Proteintech, 1:600) antibodies, respectively. Under standard protocols^32^, un-transfected or transfected LN229 and U251 GBM cells in 24-well dishes were subjected to paraformaldehyde (4%) fixation and permeability by Triton X-100 (0.1%). After the blockade with BSA (3%), the relevant antibody was diluted and added to each well. The corresponding Alexa Fluor™ 488-linked IgG (H + L) secondary antibody (#A-11008, Invitrogen) was subsequently applied at a 1:500 dilution. Nucleus staining was implemented by DAPI incubation, and images were captured and analyzed.

### Clinical specimens and immunohistochemistry (IHC) for AF127577.4-ORF

Collection and use of clinical specimens were authorized by Xinxiang Central hospital Ethic Committee. All procedures were performed in accordance with Declaration of Helsinki. All subjects signed informed consent in writing before collection of 20 fresh biopsies samples (3 normal glial tissues, 7 clinical pathological I + II grade tumors, and 10 cases III + IV grade tumors) from 17 GBM patients from our hospital. These specimens were selected in accordance with a clear pathological diagnosis.

The detection of micropeptide AF127577.4-ORF in these clinical samples was done by IHC assay based on a standard method^33^, using anti-AF127577.4-ORF antibody at a 1:200 dilution. Paraffin-embedded tissue Sects. (5 μm thickness) were sequentially subjected to incubation with anti-AF127577.4-ORF antibody, HRP secondary antibody (#GB23303, Servicebio), and DAB kit (#P0202, Beyotime, Shanghai, China) as suggested by the manufacturers.

### Calcein/PI staining

After the relevant transfection, Ln229 and U251 GBM cells were tested for cell death rate using Calcein/PI staining based on a double staining kit (#G1707, Servicebio). The death rate was scored by calculating the ratio of red/green cells.

### Proliferation analysis

The measurement of the EdU-positive cells was used for evaluation of the proliferation ability of transfected GBM cells. The Click-iT EdU-594 proliferation detection kit (#G1603, Servicebio) was applied for this assay as suggested. Transfected GBM cells were processed by 10 μM of EdU reagent for 2 h, and followed by fixation and permeabilization. Then, the working reagent containing iF594 fluorochrome (red) was added in each well. Following the 30-min incubation, cells were washed, and Hoechst 33,342 staining (blue) was done.

### Co-immunoprecipitation (Co-IP), silver staining and MS

For analysis of proteins interacted with AF127577.4-ORF, total extractions of LN229 GBM cells expressing AF127577.4-ORF-Flag peptide were processed by Co-IP using anti-Flag affinity Sepharose 4B gels (#20585ES01, Yeasen, Shanghai, China). The coprecipitated proteins were separated prior to SDS-PAGE, and the gels were stained with silver using a staining kit and accompanying protocols (#G2080, Servicebio). The extracted protein mixtures were subjected to HPLC–MS by Qinglianbio Biotechnology (Beijing, China) using L-3000 HPLC System. The RAW files were processed by homo sapiens database using Proteome Discoverer2.4 software.

To evaluate the influence of AF127577.4-ORF in ERK2/METTL3 interaction, whole lysates of vector- and AF127577.4-ORF plasmid-introduced LN229 GBM cells were subjected to Co-IP using anti-ERK2 (#ab227134, Abcam, 1:200) or anti-METTL3 (#15,073–1-AP, Proteintech, 2 μg) antibody, and the immunoprecipitates were captured by Pierce™ protein A/G agarose (Thermo Fisher Scientific). The abundance of METTL3 or ERK2 in the corresponding immunoprecipitates was gauged by WB.

### Measurement of m6A level

To assess the impact of AF127577.4-ORF in m6A methylation level, we utilized the m6A RNA methylation assay kit and protocols (#ab233491, Abcam). Briefly, in a 96-well dish, 80 μl binding solution was used before addition of 200 ng of RNA extracted from vector- or AF127577.4-ORF plasmid-introduced GBM cells, which was followed by 90 min incubation at 37 °C. m6A RNA was probed by capture antibody, detection antibody, and enhancer solution. After washing, signals were scored by reading the fluorescence value per well.

### Real-time qPCR for METTL3 mRNA analysis

Preparation of RNA from vector- and AF127577.4-ORF plasmid-introduced GBM cells was performed with RNAeasy™ RNA isolation kit (#R0024, Beyotime). cDNA was randomly primed from 300 ng extracted RNA using RevertAid RT kit (#K1691, Thermo Fisher Scientific) and subjected to real-time qPCR after a 20-fold dilution with TB Green qPCR mix (#RR430S, TaKaRa, Beijing, China) and primer sets specific for METTL3 (sense: 5′-CAAGGCTTCAACCAGGGTCT-3′, anti-sense: 5′-GGGTTGCACATTGTGTGGTC-3′). METTL3 mRNA expression was quantified by 2^−ΔΔCt^ method after normalization by a reference gene GADPH (#3790, TaKaRa).

### Cycloheximide (CHX) treatment assay

To assess the influence of AF127577.4-ORF in METTL3 protein stability by ERK, control vector- and AF127577.4-ORF plasmid-introduced GBM cells were pre-treated with or without the ERK selective activator LM22B-10 (#S6760, Selleck) at a 50 μM concentration for 2 h. Cells were then incubated with the protein synthesis inhibitor CHX ^20^ (#S7418, Selleck) at 50 μM for 0, 2, or 4 h. METTL3 protein level in treated GBM cells was quantified by WB.

### Statistical analysis

Representative data were presented from at least three repeated tests. Means were checked for difference by Prism 8 software using one-way ANOVA or Student’s *t*-test. Significance was set to *p*-value bellow 0.05.

### Supplementary Information

Supplementary Information 1.

Supplementary Information 2.

Supplementary Information 3.

Supplementary Information 4.

Supplementary Information 5.

Supplementary Information 6.

Supplementary Information 7.

Supplementary Information 8.

## Data Availability

All data analyzed in this study are included in this article and its supplementary information files.

## References

[1] Rong L, Li N, Zhang Z (2022). Emerging therapies for glioblastoma: Current state and future directions. J. Exp. Clin. Cancer Res..

[2] Minniti G, Niyazi M, Alongi F, Navarria P, Belka C (2021). Current status and recent advances in reirradiation of glioblastoma. Radiat. Oncol..

[3] Janjua TI, Rewatkar P, Ahmed-Cox A, Saeed I, Mansfeld FM, Kulshreshtha R, Kumeria T, Ziegler DS, Kavallaris M, Mazzieri R (2021). Frontiers in the treatment of glioblastoma: Past, present and emerging. Adv. Drug Deliv. Rev..

[4] Allen BK, Stathias V, Maloof ME, Vidovic D, Winterbottom EF, Capobianco AJ, Clarke J, Schurer S, Robbins DJ, Ayad NG (2015). Epigenetic pathways and glioblastoma treatment: Insights from signaling cascades. J. Cell Biochem..

[5] Dong Z, Cui H (2019). Epigenetic modulation of metabolism in glioblastoma. Semin. Cancer Biol..

[6] Yu W, Ma Y, Hou W, Wang F, Cheng W, Qiu F, Wu P, Zhang G (2021). Identification of immune-related lncRNA prognostic signature and molecular subtypes for glioblastoma. Front. Immunol..

[7] Li Z, Meng X, Wu P, Zha C, Han B, Li L, Sun N, Qi T, Qin J, Zhang Y (2021). Glioblastoma cell-derived lncRNA-containing exosomes induce microglia to produce complement C5. Promot. Chemother. Resist. Cancer Immunol. Res..

[8] Matsumoto A, Nakayama KI (2018). Hidden peptides encoded by putative noncoding RNAs. Cell Struct. Funct..

[9] Rion N, Rüegg MA (2017). LncRNA-encoded peptides: More than translational noise?. Cell Res..

[10] Wu P, Mo Y, Peng M, Tang T, Zhong Y, Deng X, Xiong F, Guo C, Wu X, Li Y (2020). Emerging role of tumor-related functional peptides encoded by lncRNA and circRNA. Mol. Cancer.

[11] Wang J, Zhu S, Meng N, He Y, Lu R, Yan GR (2019). ncRNA-encoded peptides or proteins and cancer. Mol. Ther..

[12] Zhou B, Yang H, Yang C, Bao YL, Yang SM, Liu J, Xiao YF (2021). Translation of noncoding RNAs and cancer. Cancer Lett..

[13] Tassinari V, Cesarini V, Tomaselli S, Ianniello Z, Silvestris DA, Ginistrelli LC, Martini M, De Angelis B, De Luca G, Vitiani LR (2021). ADAR1 is a new target of METTL3 and plays a pro-oncogenic role in glioblastoma by an editing-independent mechanism. Genome Biol..

[14] Visvanathan A, Patil V, Arora A, Hegde AS, Arivazhagan A, Santosh V, Somasundaram K (2018). Essential role of METTL3-mediated m(6)A modification in glioma stem-like cells maintenance and radioresistance. Oncogene.

[15] Xing J, Liu H, Jiang W, Wang L (2020). LncRNA-encoded peptide: Functions and predicting methods. Front. Oncol..

[16] Sun HL, Zhu AC, Gao Y, Terajima H, Fei Q, Liu S, Zhang L, Zhang Z, Harada BT, He YY (2020). Stabilization of ERK-phosphorylated METTL3 by USP5 Increases m(6)A methylation. Mol. Cell.

[17] Fu J, Zhu SH, Xu HB, Xu YQ, Wang X, Wang J, Kong PS (2020). Xihuang pill potentiates the anti-tumor effects of temozolomide in glioblastoma xenografts through the Akt/mTOR-dependent pathway. J. Ethnopharmacol..

[18] Sobecki M, Mrouj K, Camasses A, Parisis N, Nicolas E, Llères D, Gerbe F, Prieto S, Krasinska L, David A (2016). The cell proliferation antigen Ki-67 organises heterochromatin. Elife.

[19] González-Magaña A, Blanco FJ (2020). Human PCNA structure, function and interactions. Biomolecules.

[20] Ji W, Diao YL, Qiu YR, Ge J, Cao XC, Yu Y (2020). LINC00665 promotes breast cancer progression through regulation of the miR-379-5p/LIN28B axis. Cell Death Dis..

[21] Guo B, Wu S, Zhu X, Zhang L, Deng J, Li F, Wang Y, Zhang S, Wu R, Lu J (2020). Micropeptide CIP2A-BP encoded by LINC00665 inhibits triple-negative breast cancer progression. Embo J..

[22] Zhang M, Zhao K, Xu X, Yang Y, Yan S, Wei P, Liu H, Xu J, Xiao F, Zhou H (2018). A peptide encoded by circular form of LINC-PINT suppresses oncogenic transcriptional elongation in glioblastoma. Nat. Commun..

[23] Sendinc E, Shi Y (2023). RNA m6A methylation across the transcriptome. Mol. Cell.

[24] Garbo S, Zwergel C, Battistelli C (2021). m6A RNA methylation and beyond: The epigenetic machinery and potential treatment options. Drug Discov. Today.

[25] Xu Y, Song M, Hong Z, Chen W, Zhang Q, Zhou J, Yang C, He Z, Yu J, Peng X (2023). The N6-methyladenosine METTL3 regulates tumorigenesis and glycolysis by mediating m6A methylation of the tumor suppressor LATS1 in breast cancer. J. Exp. Clin. Cancer Res..

[26] Fang R, Chen X, Zhang S, Shi H, Ye Y, Shi H, Zou Z, Li P, Guo Q, Ma L (2021). EGFR/SRC/ERK-stabilized YTHDF2 promotes cholesterol dysregulation and invasive growth of glioblastoma. Nat. Commun..

[27] He B, Wang J (2021). METTL3 regulates hippocampal gene transcription via N6-methyladenosine methylation in sevoflurane-induced postoperative cognitive dysfunction mouse. Aging.

[28] Zhang M, Yang C, Ruan X, Liu X, Wang D, Liu L, Shao L, Wang P, Dong W, Xue Y (2022). CPEB2 m6A methylation regulates blood-tumor barrier permeability by regulating splicing factor SRSF5 stability. Commun Biol.

[29] Vizcaíno JA, Côté RG, Csordas A, Dianes JA, Fabregat A, Foster JM, Griss J, Alpi E, Birim M, Contell J (2013). The PRoteomics IDEntifications (PRIDE) database and associated tools: Status in 2013. Nucleic Acids Res..

[30] Dennis G, Sherman BT, Hosack DA, Yang J, Gao W, Lane HC, Lempicki RA (2003). DAVID: Database for annotation, visualization, and integrated discovery. Genome Biol..

[31] Pierce BG, Hourai Y, Weng Z (2011). Accelerating protein docking in ZDOCK using an advanced 3D convolution library. PLoS One.

[32] Park HJ, Ji P, Kim S, Xia Z, Rodriguez B, Li L, Su J, Chen K, Masamha CP, Baillat D (2018). 3' UTR shortening represses tumor-suppressor genes in trans by disrupting ceRNA crosstalk. Nat. Genet..

[33] Korpal M, Ell BJ, Buffa FM, Ibrahim T, Blanco MA, Celià-Terrassa T, Mercatali L, Khan Z, Goodarzi H, Hua Y (2011). Direct targeting of Sec23a by miR-200s influences cancer cell secretome and promotes metastatic colonization. Nat. Med..

