# Supplementary material for: Micropeptide AF127577.4-ORF hidden in a lncRNA diminishes glioblastoma cell proliferation via the modulation of ERK2/METTL3 interaction
Source: Sci Rep. 2024 May 27;14:12090. doi: 10.1038/s41598-024-62710-y (PMC11130299; doi:10.1038/s41598-024-62710-y)
Supplement: Supplementary file 1 — Supplementary Information 1. [file 41598_2024_62710_MOESM1_ESM.docx]

Figure 2A

Marker: 10kDa, 18kDa, 23kDa, 30kDa, 42kDa, 55kDa, 75kDa, 110kDa, 140kDa, 200kDa

Flag


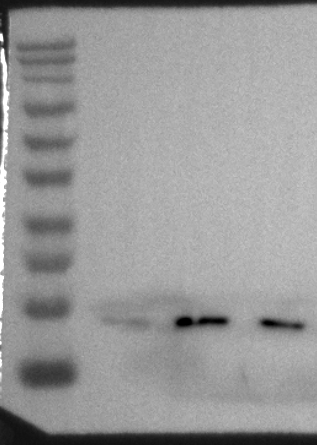


Figure 2E

Marker: 20kDa, 30kDa, 40kDa, 50kDa, 60kDa, 80kDa, 120kDa

Flag-LN229 (left), U251 (right)


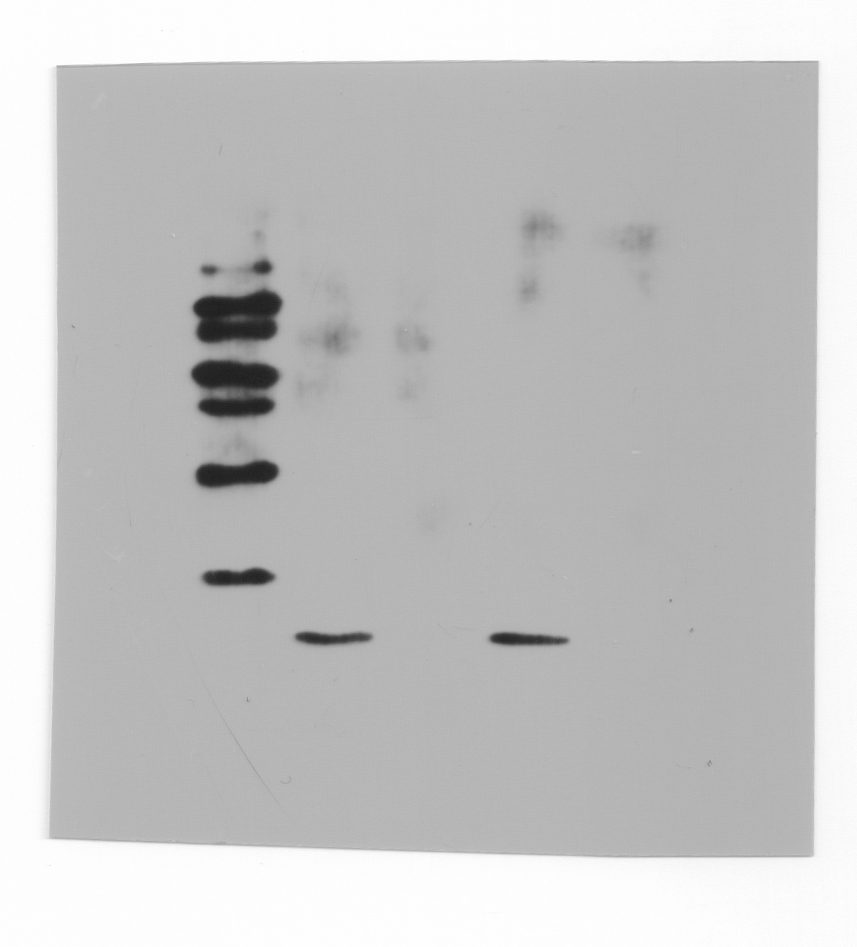


Figure 2J

AF127577.4-ORF GAPDH

**
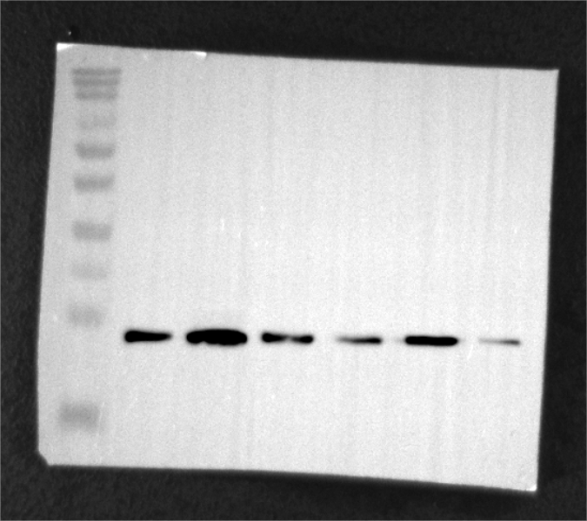

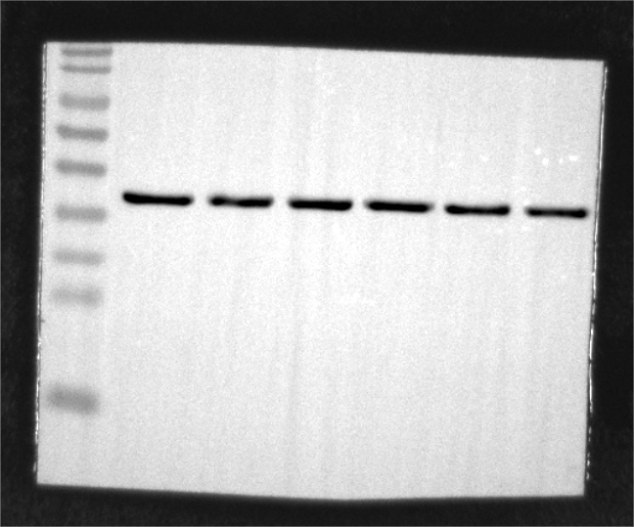
**

Marker: 10kDa, 18kDa, 23kDa, 30kDa, 42kDa, 55kDa, 75kDa, 110kDa, 140kDa, 200kDa

Figure 5A

**
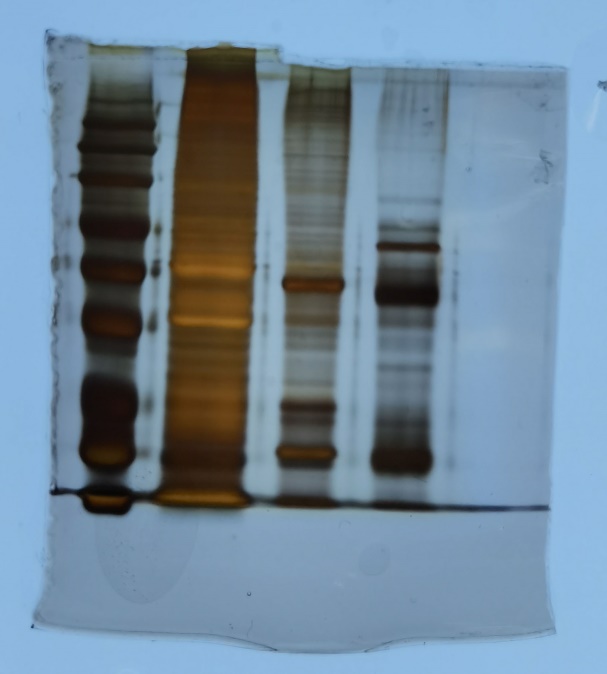
**

Marker: 10kDa, 18kDa, 23kDa, 30kDa, 42kDa, 55kDa, 75kDa, 110kDa, 140kDa, 200kDa

METTL3


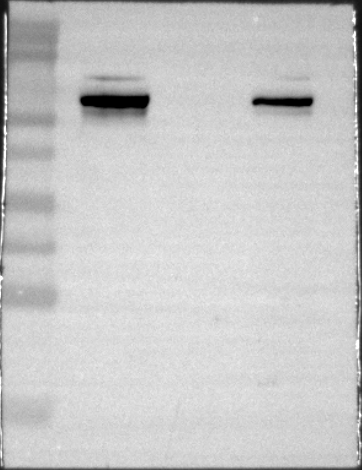


ERK2


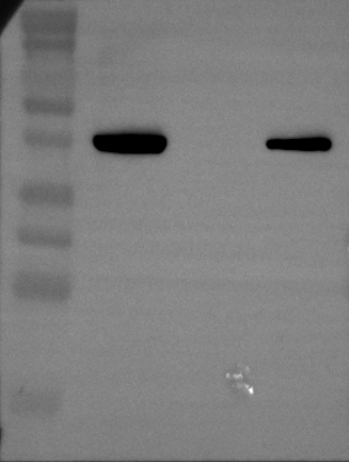


Figure 6B

Marker: 10kDa, 18kDa, 23kDa, 30kDa, 42kDa, 55kDa, 75kDa, 110kDa, 140kDa, 200kDa

METTL3


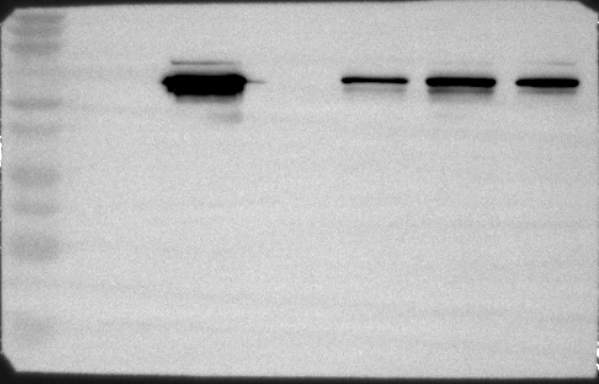


ERK2


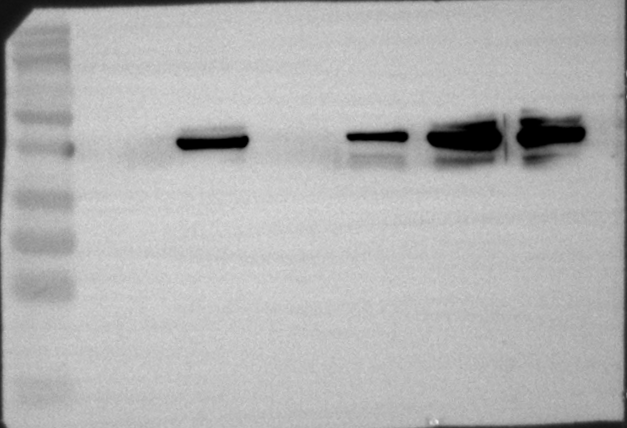


Figure 6D

Marker: 10kDa, 18kDa, 23kDa, 30kDa, 42kDa, 55kDa, 75kDa, 110kDa, 140kDa, 200kDa

U251-p-ERK


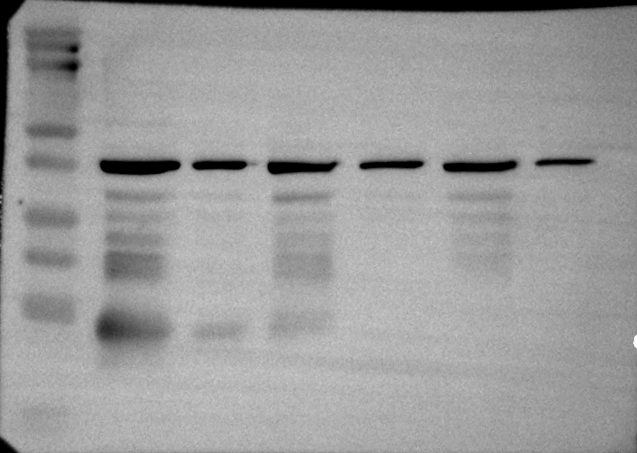


U251-ERK2


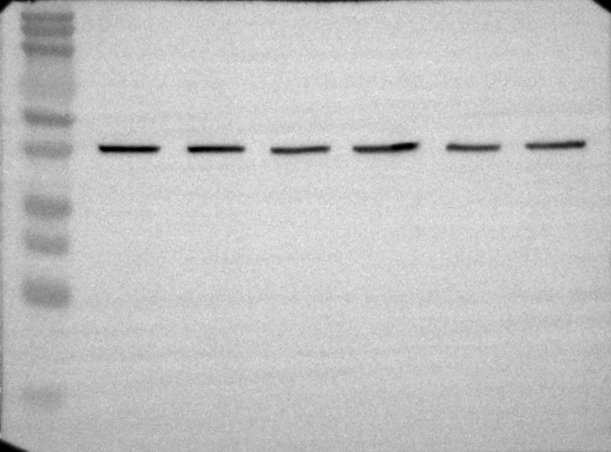


LN229-p-ERK


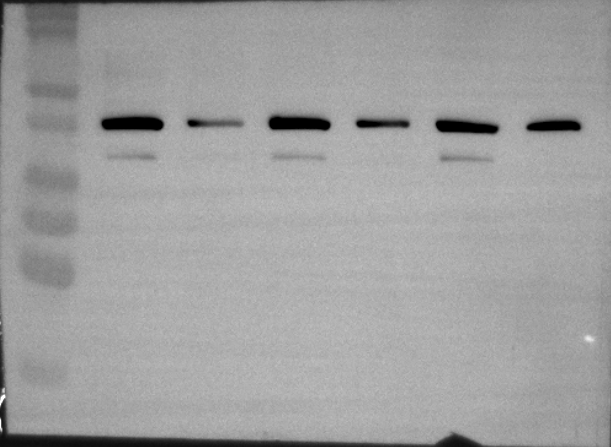


LN229-ERK2


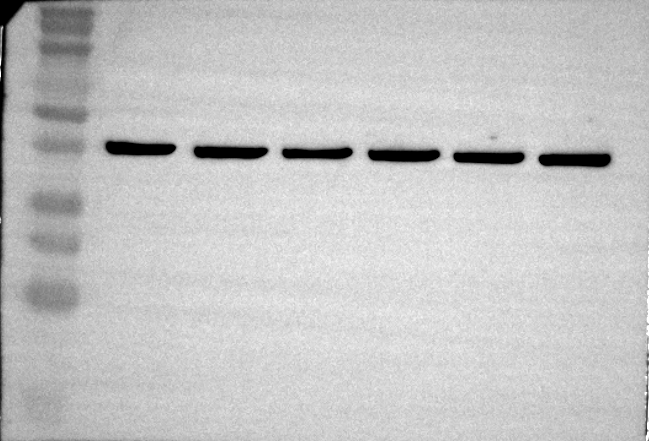


Figure 6E

Figure 6F-LN229

p-ERK


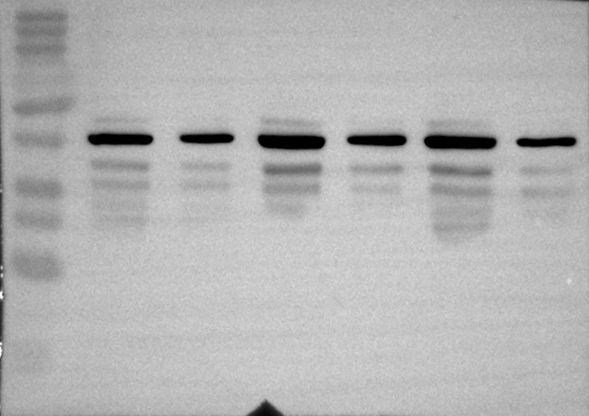


ERK2


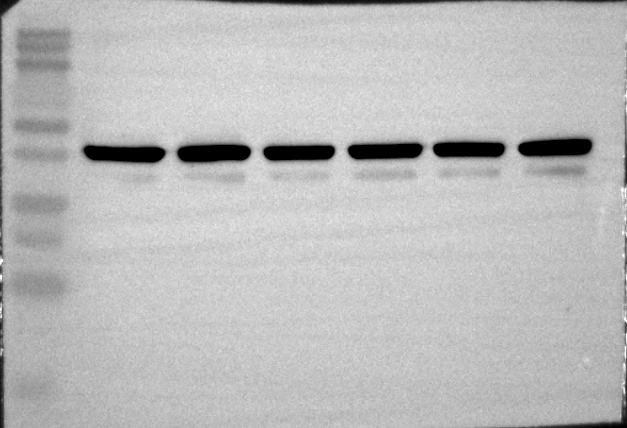


METTL3


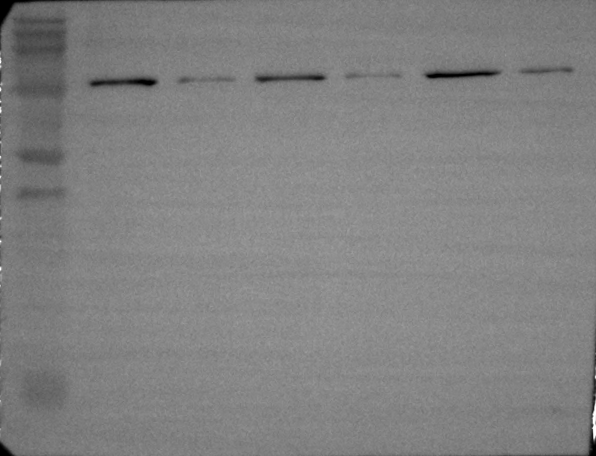


GAPDH


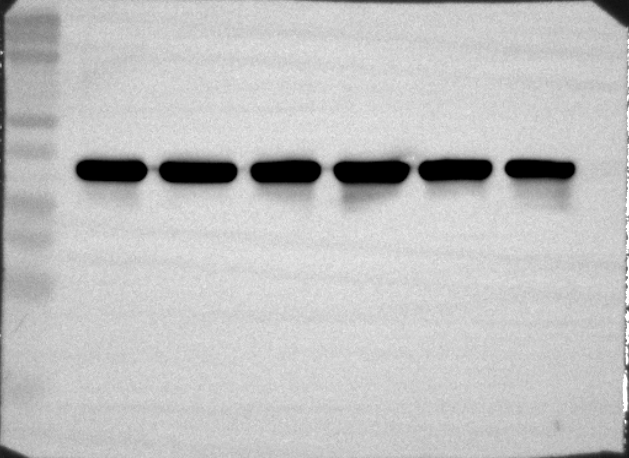


Marker: 10kDa, 18kDa, 23kDa, 30kDa, 42kDa, 55kDa, 75kDa, 110kDa, 140kDa, 200kDa

Figure 6F-U251

p-ERK


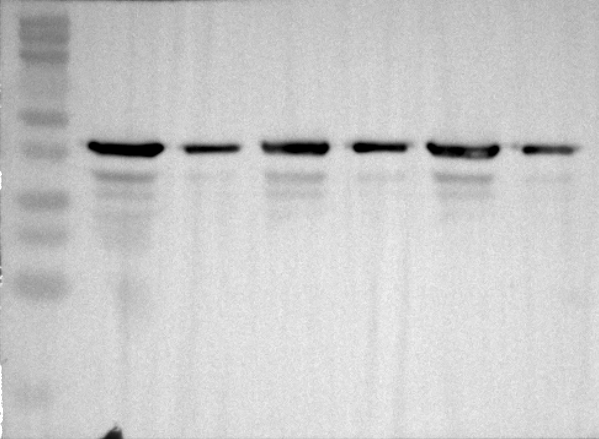


ERK2


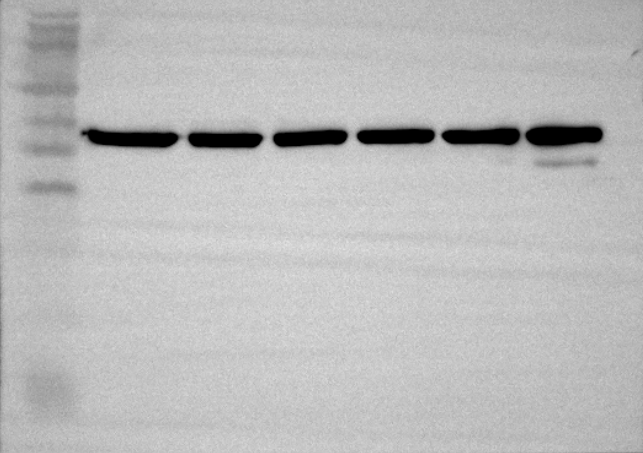


GAPDH


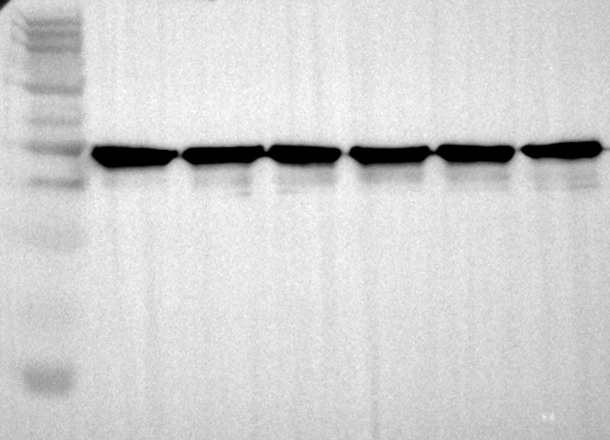


METTL3


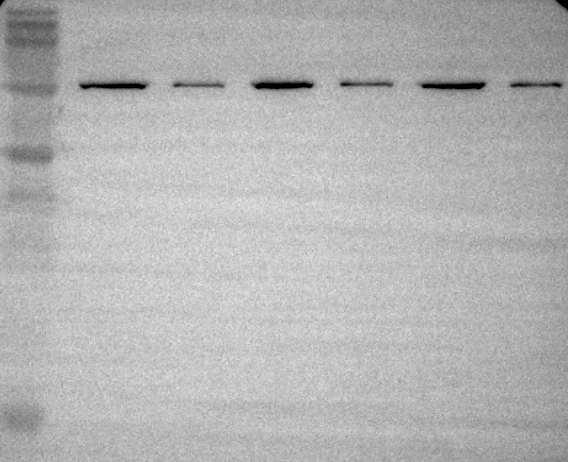


Marker: 10KDa, 15KD, 20KDa, 30KDa, 40KDa, 55KDa, 65KDa, 90KDa, 110KDa, 140KDa, 200KDa

Figure 6H

LN229-METTL3


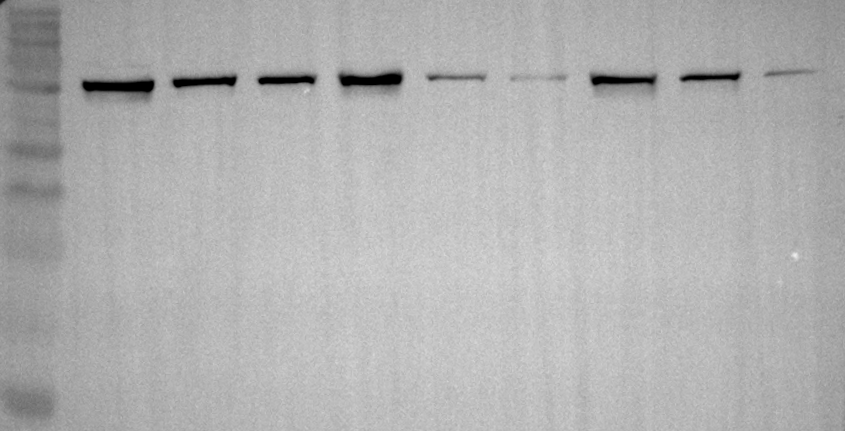


LN229-GAPDH


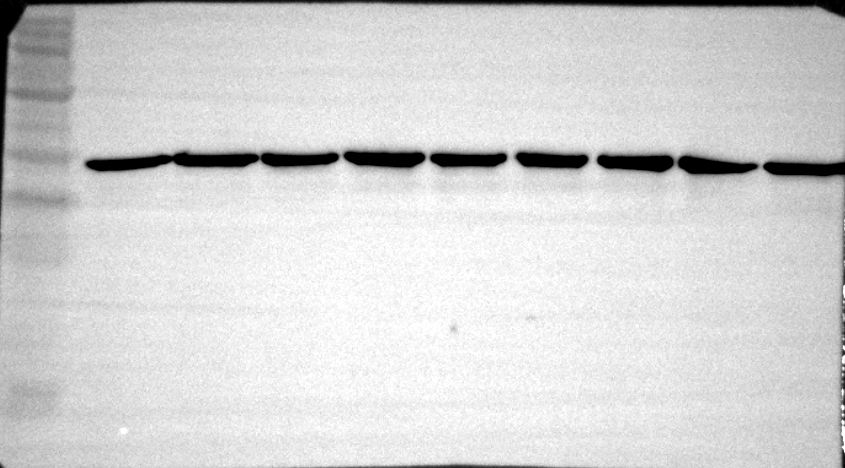


U251-METTL3


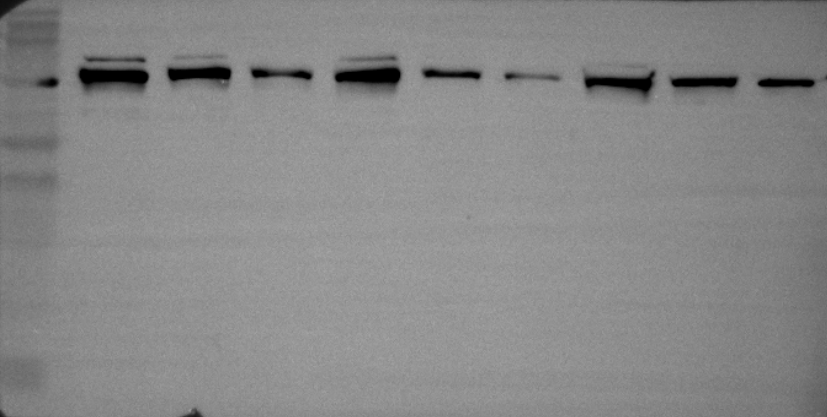


U251-GAPDH


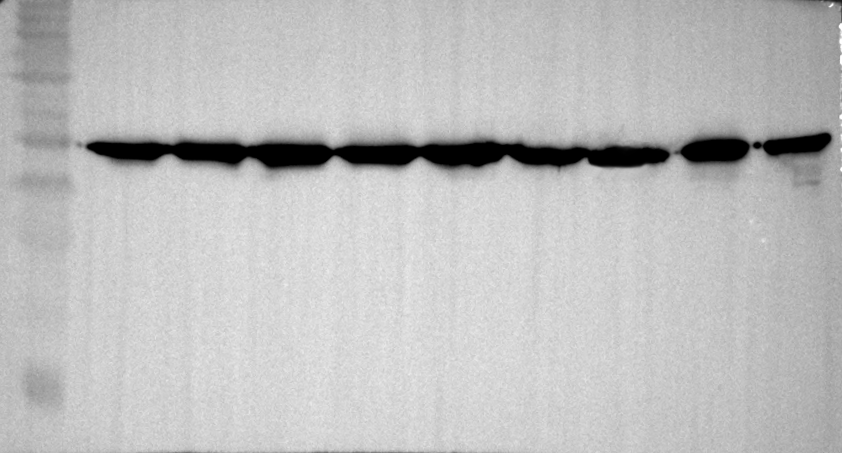


Marker: 10KDa, 15KD, 20KDa, 30KDa, 40KDa, 55KDa, 65KDa, 90KDa, 110KDa, 140KDa, 200KDa
