# Supplementary figures and images for: Micropeptide AF127577.4-ORF hidden in a lncRNA diminishes glioblastoma cell proliferation via the modulation of ERK2/METTL3 interaction
Source: Sci Rep. 2024 May 27;14:12090. doi: 10.1038/s41598-024-62710-y (PMC11130299; doi:10.1038/s41598-024-62710-y)

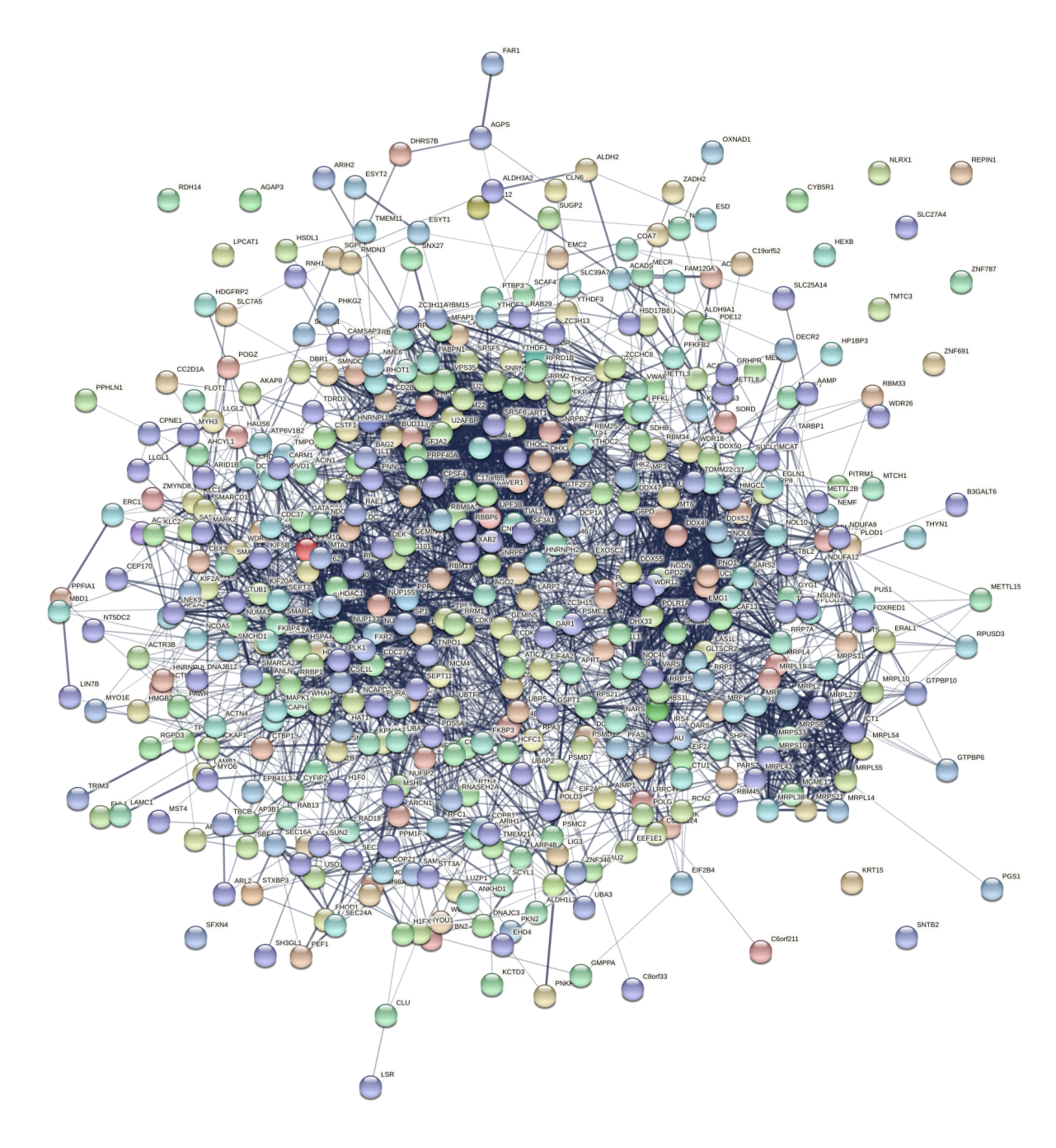

Supplement: Supplementary file 2 — Supplementary Information 2. [file 41598_2024_62710_MOESM2_ESM.tif]
